# Clinical Correlates of Health Preference and Generic Health-Related Quality of Life in Patients with Colorectal Neoplasms

**DOI:** 10.1371/journal.pone.0058341

**Published:** 2013-03-13

**Authors:** Carlos K. H. Wong, Cindy L. K. Lam, Jensen T. C. Poon, Dora L. W. Kwong

**Affiliations:** 1 Department of Family Medicine and Primary Care, The University of Hong Kong, Hong Kong, Hong Kong; 2 Department of Surgery, The University of Hong Kong, Hong Kong, Hong Kong; 3 Department of Clinical Oncology, The University of Hong Kong, Hong Kong, Hong Kong; Tehran University of Medical Sciences, Iran (Islamic Republic Of)

## Abstract

**Background:**

The aims of the study were to assess the health preference and health-related quality of life (HRQOL) in patients with colorectal neoplasms (CRN), and to determine the clinical correlates that significantly influence the HRQOL of patients.

**Methods:**

Five hundred and fifty-four CRN patients, inclusive of colorectal polyp or cancer, who attended the colorectal specialist outpatient clinic at Queen Mary Hospital in Hong Kong between October 2009 and July 2010, were included. Patients were interviewed with questionnaires on socio-demographic characteristics, and generic and health preference measures of HRQOL using the SF-12 and SF-6D Health Surveys, respectively. Clinical information on stage of disease at diagnosis, time since diagnosis, primary tumour site was extracted from electronic case record. Mean HRQOL and health preference scores of CRN patients were compared with age-sex matched controls from the Chinese general population using independent t-test. Multiple linear regression analyses were conducted to explore the associations of clinical characteristics with HRQOL measures with the adjustment of socio-demographic characteristics.

**Results:**

Cross-sectional data of 515 eligible patients responded to the whole questionnaires were included in outcome analysis. In comparison with age-sex matched normative values, CRN patients reported comparable physical-related HRQOL but better mental-related HRQOL. Amongst CRN patients, time since diagnosis was positively associated with health preference score whilst patients with rectal neoplasms had lower health preference and physical-related HRQOL scores than those with sigmoid neoplasms. Health preference and HRQOL scores were significantly lower in patients with stage IV colorectal cancer than those with other less severe stages, indicating that progressive decline from low-risk polyp to stage IV colorectal cancer was observed in HRQOL scores.

**Conclusion:**

In CRN patients, a more advanced stage of disease was associated with worse HRQOL scores. Despite potentially adverse effect of disease on physical-related HRQOL, the mental-related HRQOL of CRN patients were better than that of Chinese general population.

## Introduction

Colorectal neoplasms (CRN) represent a wide spectrum of disease transition from precancerous colorectal polyps to colorectal cancer. In the past, disease management was principally evaluated by the effectiveness in prolonging survival and reducing disease-related complications and presentation of symptoms. The maintenance of health-related quality of life (HRQOL) following screening and treatment has driven an important new direction in research and clinical practice for patients with CRN. The assessment of HRQOL on CRN has also increased the understanding in the treatment efficiency and effectiveness, and service needs of rehabilitation[1] for the interest of patients and clinicians, and subsequently facilitated the clinical decision makings[2].

There is no consensus on whether the HRQOL of CRN patients was influenced by clinical factors. For instance, there have been few attempts to characterize the associations of HRQOL with the cancer stages and the time since cancer diagnosis [3]–[5]. Evidence from the US studies did not demonstrate any associations between HRQOL and time since diagnosis in colorectal cancer patients[3]–[5]. Significant associations between HRQOL and stage at cancer diagnosis did not exist in colorectal cancer survivors[3], [4] but did happen in mixture of survivors and non-survivors[5]. Such comparative data on the HRQOL of colorectal cancer patients with different cancer stages were limited to the US population, which might not be transferable to Asian or Chinese populations. Furthermore, no study has investigated the HRQOL of patients with colorectal polyps. The health preference score of colorectal polyp patients is contributed to the economic evaluation that informs health policy making. The cost-effectiveness analysis is one type of economic evaluation, and assesses the health outcomes in terms of quality-adjusted life years (QALYs)[6]. The QALYs is calculated by the sum of the life years weighted by the health preference scores, anchoring on a scale from zero (death) to one (full health), of each individual staying at specific health state. Therefore, the health preference of colorectal polyp patients enables the calculation of QALYs to compare no screening against alternative colorectal cancer screening strategies in economics evaluation.

The aims of this study were: to assess the HRQOL, as measured by Short-Form 12-Item (SF-12) and 6-dimensions (SF-6D) Health Survey, of patients with different stages of CRN; and to investigate which clinical factors were related to the HRQOL of patients with CRN, after adjustment for the socio-demographic characteristics.

## Materials and Methods

### Ethics Statement

Ethical approval was obtained from The University of Hong Kong/ Hospital Authority Hong Kong West Cluster institutional review board (HKU/HA HKW IRB #UW 09-391).

The study was conducted using HRQOL instruments (generic and health preference measures) to evaluate the HRQOL of patients with CRN who attended the colorectal specialist outpatient clinic at the Queen Mary Hospital in Hong Kong from October 2009 to July 2010. The inclusion criteria were that the adults patients had known stage of disease classification based on the colorectal neoplasm screening surveillance guideline[7] and the American Joint Committee on Cancer (AJCC) staging classification system for colorectal cancer[8]: 1) Low-risk polyps group (patients with ≤2 adenomas or 3–4 adenomas all of which were not larger than 1 cm); 2) High-risk polyps group (patients with ≥5 adenomas or with ≥3 adenomas at least one of which was larger than 1 cm); 3) Stage I; 4) Stage II; 5) Stage III and 6) Stage IV. Those who were classified as unknown stage of disease or primary tumour site when it was unspecified in the medical record, those who was diagnosed with CRN for less than six months, those who had an expected life expectancy of less than six months, and those who had low literacy and cognitive impairment were excluded from this study. A total of 698 patients were assessed for eligibility and 600 eligible patients gave consent to enroll in the longitudinal study administering questionnaire at baseline, six months and twelve months later. The baseline data from the longitudinal study formed the basis of the current study. Five hundred and fifty-four (response rate: 79.4%) patients completed the questionnaire either by face-to-face interviews at the outpatient clinic or by telephone interviews within one month after the first contact for this research purpose at the outpatient clinic. Details of recruitment procedure have been described in authors' previous publications[9]–[14].

Matched samples from an external study were used for comparison of HRQOL between CRN and the Chinese general population[15]. Age-sex matched controls of 515 samples were randomly selected from a larger representative 2533 samples of Chinese general population, who were administered SF-12v2 health survey in the primary care service utilization study in 2010 by trained interviewers.

### HRQOL Measures

This study administered the traditional Chinese version of SF-12v2 and SF-6D Health Surveys, which have been validated[16]–[19] and normed[15] in the general Chinese population in Hong Kong.

The Chinese (HK) SF-12v2 Health Survey is a 12-item generic HRQOL measure consisted of eight subscales: Physical functioning (PF), Role physical (RP), Bodily pain (BP), General health (GH), Vitality (VT), Social functioning (SF), Role emotional (RE) and Mental health (MH); and two composite summary scores: Physical Component Summary (PCS) and Mental Component Summary (MCS) scores. All subscales and summary scores were ranged from 0-100. The higher the SF-12 score, the better the HRQOL outcome. Such generic measure of HRQOL is used for measuring HRQOL of the general population including individuals living with any health condition, and subsequently enabling the head-to-head comparison between patients with CRN and population norm.

The Chinese (HK) SF-6D Health Survey, a subset of SF-12 [20] and SF-36 [21] Health Surveys, is a 6-item health preference measure ranging from 0(dead) to 1(perfect health). The SF-6D health preference scores are calculated by converting health state into a single index weighted summation from the SF-6D preference weight coefficients based on published scoring algorithm for Hong Kong population [22]. The theoretical range of SF-6D is from 0.315 (worst possible health state) to 1 (perfect health) based on Hong Kong scoring algorithm.

### Patient Characteristics

Self-reported socio-demography including age, gender, marital status (Married/Non-married), educational level (No formal schooling/Primary/Secondary/Tertiary or above), household monthly income (≤HKD$20,000/>HKD$20,000, pegged at an exchange rate of USD$1 = HKD$7.8), occupation status (Working/Non-working), smoking (Ever had/Never had) and drinking (Ever had/Never had) were collected by interviewers via telephone or face-to-face interview. Clinical characteristics including stage of colorectal neoplasm at diagnosis (Polyp: Low-risk and High-risk; Colorectal cancer: Stage I, II, III and IV), time since diagnosis (months), family history of colorectal cancer (Parents or sibling/No), primary tumour site (Colon/Rectum/Sigmoid), colorectal cancer relapse, active cancer treatment and stoma (Present/Absent) were retrieved from the electronic medical records. The later three clinical characteristics were only applicable to 380 patients diagnosed with colorectal cancer.

### Statistical Analysis

Socio-demographic and clinical characteristics were described overall, and by tumour stage at initial diagnosis. Chi-square or one-way analysis of variance (ANOVA) tests were used to assess the differences in categorical and continuous variables, respectively, according to the stage at diagnosis. The complete data set (n = 515), excluding 38 patients with unknown staging/primary site (n = 12) or missing values in any socio-demographic and clinical factors (n = 26), was used for further analysis of HRQOL by mean comparisons and regression analyses. HRQOL scores were determined for each stage, and the differences between stages were tested by one-way ANOVA with Tukey's post hoc test. The statistical power calculation was based on detecting a difference of at least 5 points in the SF-12 scores (as extrapolated from minimum clinically important difference for the SF-36 subscale scores[23]) between colorectal polyp and cancer groups, with a standard deviation of 10 for the Chinese population[16] and 80% power at type I error of 5%. Estimated sample size of 135 was obtained from each group, in total of 270 samples required in this study.

Normative values of SF-12v2 subscale and summary scores were extracted from 515 matched controls of 2533 samples reported in general population survey in Hong Kong[15]. Mean HRQOL scores of CRN were tested against the general population norms using independent t-test.

Health preference and two SF-12v2 summary (PCS and MCS) scores were used as dependent variables in the regression analyses. Multivariate linear regression analyses were modeled to determine the effect of clinical factors on dependent variables, controlling for the effects of socio-demographic factors. No forward or backward selection procedure was applied for each regression analysis so all variables were entered in one step only. Each significant regression coefficients are presented with standard error, a 95% confidence interval, level of multicollinearity measured by tolerance and variance inflation factors (VIF). The multicollinearity occurred if the tolerance statistic was less than 0.1 or VIF was greater than 10 as a rule of thumb. The *R^2^* and adjusted *R^2^* representing the total variances of dependent variables explained were reported together with the corresponding regression analyses. Normality of residuals was examined to check the model validity by residual plot.

The SPSS Windows 20.0 program (IBM SPSS Inc., Chicago IL, USA) was used for all statistical analyses. A P-value of <0.05 was interpreted as statistically significant in all tests.

## Results

### Sample Characteristics

Table 1 shows the socio-demographic characteristics of the study subjects. The majority of patients were male (58%), married (75.2%), not working (75.6%), low household monthly income (83.6%), non-smokers (73.1%) and non-drinkers (72.3%). The clinical characteristics of subjects are listed in Table 2. Ninety-three (16.8%) and 72 (13.0%) subjects were classified as low-risk and high-risk polyp group, respectively. Eighty-three (15.0%) patients were subsequently diagnosed with stage I, 101 (18.3%) with stage II, 114 (20.6%) with stage III and 82 (14.8%) with stage IV. Of those patients with colorectal cancer, 36 (6.5%) reported relapse which was more common in advanced stage than in early stage of cancer. The mean time since diagnosis was 46.7 months (SD: 55), ranged from 6 to 377 months, with a shorter time since diagnosis for Stage IV cancer. Thirty-eight point three percent of primary tumours were located in the colon, 39.8% were at the rectum and 21% were at the sigmoid colon. Most patients (86.2%) did not have stoma at the time of assessment, and most patients (76.9%) finished treatment such as adjuvant and palliative chemotherapy and/or radiotherapy.

**Table 1:** Socio-Demographic Characteristics of Study Subjects by Colorectal Neoplasm Staging.

			Polyp (n = 165)		Colorectal Cancer AJCC Staging (n = 381)				
	Total (n = 554)	P-value	Low Risk (n = 93)	High Risk (n = 72)	StageI (n = 84)	StageII (n = 101)	StageIII (n = 114)	StageIV (n = 82)	Unknown (n = 8)
Age (Year,mean±SD)*	63.3±11.3	<0.001* ‡	59.7±10.4	63.3±12.4	66.8±10.3	66±10.7	61.7±11	62.6±12.1	60.6±10.8
Sex (%)		0.015†							
Male	58.1%		58.1%	77.8%	47.6%	57.4%	56.1%	56.1%	50.0%
Female	41.9%		41.9%	22.2%	52.4%	42.6%	43.9%	43.9%	50.0%
Education Level (%)		0.379							
No formal school	10.5%		6.5%	5.6%	9.5%	15.8%	12.3%	12.2%	0.0%
Primary	35.4%		28.0%	43.1%	40.5%	37.6%	31.6%	31.7%	62.5%
Secondary	41.3%		48.4%	38.9%	40.5%	36.6%	42.1%	41.5%	37.5%
Tertiary	12.8%		17.2%	12.5%	9.5%	9.9%	14.0%	14.6%	0.0%
Marital Status (%)		0.063							
Married	75.6%		81.7%	81.7%	61.9%	75.2%	76.3%	76.8%	75.0%
Not married	24.4%		18.3%	18.3%	38.1%	24.8%	23.7%	23.2%	25.0%
Currently Working (%)†		<0.001†							
Yes	24.4%		37.6%	27.8%	17.9%	18.8%	29.8%	11.0%	37.5%
No	75.6%		62.4%	72.2%	82.1%	81.2%	70.2%	89.0%	62.5%
Household Monthly Income (%)		<0.001†							
≤HKD$20,000	83.7%		68.5%	80.9%	89.3%	92.0%	80.4%	91.0%	87.5%
>HKD$20,000	16.3%		31.5%	19.1%	10.7%	8.0%	19.6%	9.0%	12.5%
Smoking (%)		0.042†							
Ever had	26.9%		26.9%	36.1%	21.4%	31.7%	16.7%	31.7%	37.5%
Never had	73.1%		73.1%	63.9%	78.6%	68.3%	83.3%	68.3%	62.5%
Drinking (%)		0.101							
Ever had	27.4%		31.2%	30.6%	16.7%	27.7%	23.7%	34.1%	50.0%
Never had	72.6%		68.8%	69.4%	83.3%	72.3%	76.3%	65.9%	50.0%

Note: AJCC = American Joint Committee on Cancer

* Significant difference between colorectal neoplasm staging (except unknown stage)by One-Way ANOVA

† Significant difference between colorectal neoplasm staging (except unknown stage)by Chi-square Test

‡ Significant difference between colorectal neoplasm staging (except unknown stage) by Tukey's Post-hoc multiple comparisons. StageI, StageII > Low Risk

**Table 2:** Clinical Characteristics of Study Subjects by Colorectal Neoplasm Staging.

			Polyp (n = 165)		Colorectal Cancer AJCC Staging (n = 381)				
	Total (n = 554)	P-value	Low Risk (n = 93)	High Risk (n = 72)	StageI (n = 84)	StageII (n = 101)	StageIII (n = 114)	StageIV (n = 82)	Unknown (n = 8)
Primary Site (%)‡		<0.001‡							
Colon	38.3%		55.9%	56.9%	20.2%	37.6%	28.9%	35.4%	25.0%
Rectum	39.9%		20.4%	23.6%	59.5%	44.6%	48.2%	36.6%	62.5%
Sigmoid	20.9%		20.4%	19.4%	19.0%	17.8%	22.8%	28.0%	0.0%
Other*	0.9%		3.2%	0.0%	1.2%	0.0%	0.0%	0.0%	12.5%
Family History of CRC (%)	18.5%	0.241	21.5%	25.7%	15.5%	15.0%	14.0%	20.7%	37.5%
Month of Last Diagnosis (Mean±SD)†	46.7±55.8	<0.001† ¶	34.4±36.0	29.7±43.5	68.5±65.8	66.7±63.3	44.1±55.8	25.8±28.6	131.0±131.7
Chronic co-morbidities‡		0.001‡							
Present	64.8%		71.0%	61.1%	76.2%	72.3%	60.5%	47.6%	50.0%
Absent	35.2%		29.0%	38.9%	23.8%	27.7%	39.5%	52.4%	50.0%
CRC Relapsed (%)§	9.4%	<0.001§	NA	NA	2.4%	4.0%	5.3%	29.3%	0.0%
Current CRC Treatment (%)§		<0.001§							
No	76.9%		NA	NA	100.0%	96.0%	78.9%	26.8%	80.0%
Yes	23.1%		NA	NA	0.0%	4.0%	21.1%	73.2%	20.0%
Stoma (%)§		0.015§							
Present	13.7%		NA	NA	14.3%	9.9%	11.4%	18.3%	37.5%
Absent	86.3%		NA	NA	85.7%	90.1%	88.6%	81.7%	25.0%

Note: AJCC = American Joint Committee on Cancer

* Other included those who had unknown or double primary site.

† Significant difference between colorectal neoplasm staging (except unknown stage) by One-Way ANOVA

‡ Significant difference between colorectal neoplasm staging (except unknown stage) by Chi-square Test

§ Significant difference between colorectal cancer staging (except unknown stage) by Chi-square Test

¶ Significant difference between colorectal neoplasm staging (except unknown stage) by Tukey's Post-hoc multiple comparisons. StageI, StageII > Low Risk, High Risk, StageIII, StageIV

### Comparisons among Stage of Disease and with General Population

**Figure 1:**
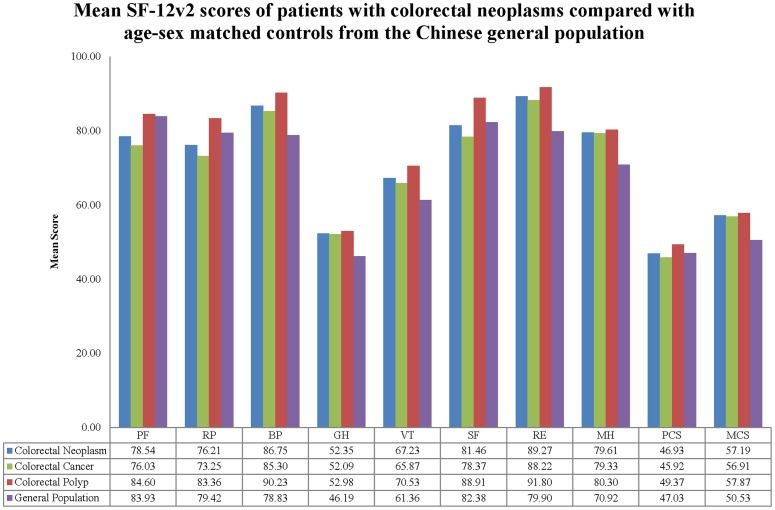
Mean SF-12v2 scores of patients with colorectal neoplasms compared with age-sex matched controls from the Chinese general population.

Table 3 and Figure 1 presents the subscale and overall mean SF-12 subscale scores for CRN patients compared with general population. CRN patients reported statistically worse scores for the physical aspects of HRQOL (PF, RP and PCS) compared with general population. For other aspects of HRQOL, CRN patients reported statistically better scores for BP, GH, VT, RE, MH and MCS than general population.

**Table 3:** Descriptive Statistics (Mean and SD) of HRQOL Scores for Patients with Colorectal Neoplasms Compared with Age-Sex Matched Controls from Chinese General Population.

	Age-Sex Matched Controls (N = 515)		Colorectal Neoplasms (N = 515)		Difference (Matched Controls - Colorectal Neoplasms)			Difference (Matched Controls - Colorectal polyp)			Difference (Matched Controls - Colorectal cancer)		
Measure/Scale	Mean±SD	95% C.I.	Mean±SD	95% C.I.	Mean±SD	95% C.I.	P-value	Mean±SD	95% C.I.	P-value	Mean±SD	95% C.I.	P-value
**SF-12v2**													
PF	83.9±25.1	(81.8,86.1)	78.5±29.6	(76.0,81.1)	5.39±1.71	(2.04,8.74)	0.002*	−1.46±2.37	(−6.10,3.19)	0.538	7.11±1.91	(3.37,10.85)	<0.001*
RP	79.4±25.3	(77.2,81.6)	76.2±27.6	(73.8,78.6)	3.20±1.65	(−0.04,6.44)	0.053	−5.07±2.34	(−9.66, −0.48)	0.031*	5.04±1.87	(1.37,8.71)	0.007*
BP	78.8±25.9	(76.6,81.1)	86.7±22.9	(84.8,88.7)	−7.91±1.52	(−10.90, −4.93)	<0.001*	−12.49±2.32	(−17.04, −7.93)	<0.001*	−7.56±1.75	(−10.99, −4.12)	<0.001*
GH	46.2±28.6	(43.7,48.7)	52.3±25.8	(50.1,54.6)	−6.16±1.70	(−9.49, −2.83)	<0.001*	−7.87±2.49	(−12.75, −2.98)	0.002*	−6.97±1.86	(−10.63, −3.32)	<0.001*
VT	61.4±28.3	(58.9,63.8)	67.2±19.7	(65.5,68.9)	−5.87±1.52	(−8.86, −2.89)	<0.001*	−10.68±2.46	(−15.51, −5.86)	<0.001*	−6.02±1.74	(−9.42, −2.61)	<0.001*
SF	82.4±25.1	(80.2,84.5)	81.5±28.6	(79.0,83.9)	0.92±1.67	(−2.36,4.21)	0.582	−6.90±2.33	(−11.47, −2.33)	0.003*	3.64±1.90	(−0.08,7.36)	0.055
RE	79.9±22.6	(77.9,81.9)	89.3±19.4	(87.6,91.0)	−9.37±1.31	(−11.94, −6.79)	<0.001*	−12.78±1.98	(−16.68, −8.88)	<0.001*	−9.20±1.50	(−12.15, −6.25)	<0.001*
MH	70.9±19.3	(69.3,72.6)	79.6±15.6	(78.3,81.0)	−8.69±1.09	(−10.84, −6.54)	<0.001*	−9.37±1.66	(−12.62, −6.12)	<0.001*	−8.40±1.23	(−10.81, −5.99)	<0.001*
PCS	47.0±11.5	(46.0,48.0)	46.9±10.6	(46.0,47.8)	0.10±0.69	(−1.25,1.45)	0.885	−2.76±1.03	(−4.79, −0.73)	0.002*	0.70±0.78	(−0.84,2.23)	0.369
MCS	50.5±10.4	(49.6,51.4)	57.2±8.0	(56.5,57.9)	−6.66±0.58	(−7.80, −5.53)	<0.001*	−7.48±0.89	(−9.22, −5.74)	<0.001*	−6.52±0.64	(−7.79, −5.26)	<0.001*

Note:

SF-12 subscales: PF = physical functioning; RP = role physical; BP = bodily pain; GH = general health; VT = vitality; SF = social functioning; RE = role emotional; MH = mental health; PCS = Physical Composite Summary; MCS = Mental Composite Summary

Higher score represents a higher level of functioning or better quality of life

* Significant difference between samples from our study and general population surveys by independent t-test

**Figure 2:**
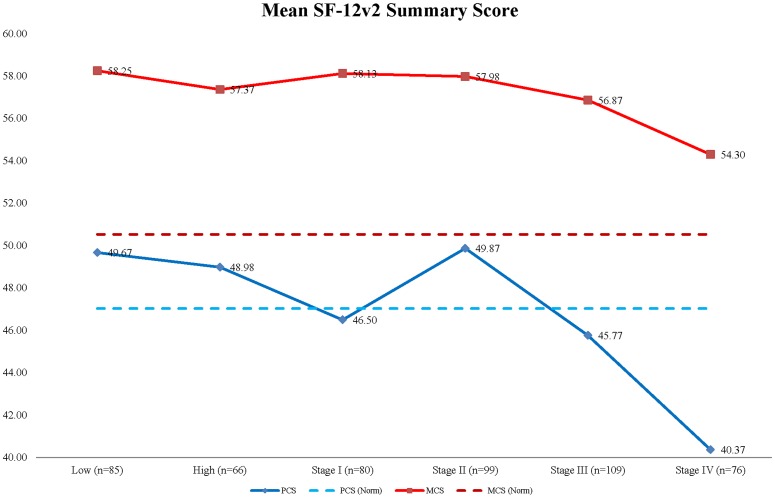
Mean SF-12v2 summary scores by stage of disease.

**Figure 3:**
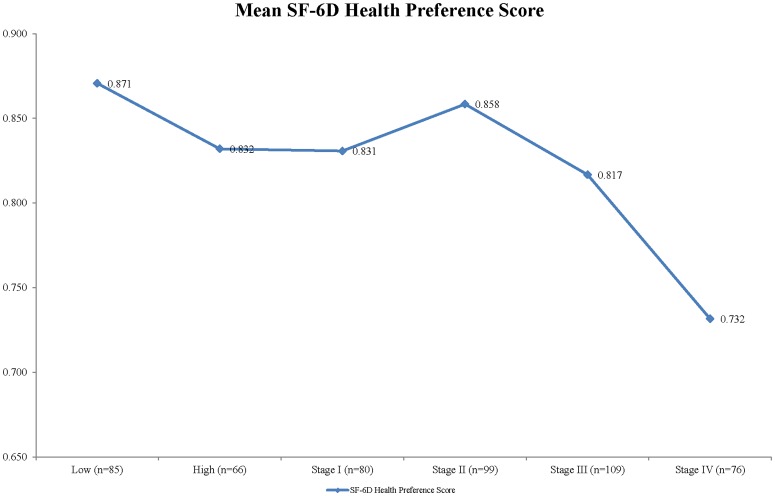
Mean SF-6D health preference score by stage of disease.

Mean HRQOL scores in relation to stage of disease at diagnosis are presented in Table 4, Figure 2 and Figure 3. Patients with metastatic colorectal cancer had the lowest HRQOL, with significantly lower HRQOL scores than low-risk polyp and early stages of colorectal cancer. There was an unexpected increase trend in the MCS and health preference scores from early to late stages of cancer although the differences were not statistically significant. Health preference scores of stage II patients were generally higher than those of patients with late stages of cancer.

**Table 4:** Descriptive Statistics (Mean and SD) of Questionnaire Scores at Baseline by CRN Groups

		Colorectal Neoplasm Staging						
Measure/Scale	Total (n = 515)	Low Risk (n = 85)	High Risk (n = 66)	Stage I (n = 80)	Stage II (n = 99)	Stage III (n = 109)	Stage IV (n = 76)	Multiple Comparison*
**SF-12**								
PF	78.5±29.6	85.0±24.8	84.1±25.8	77.2±30.3	85.1±25.2	75.7±30.7	63.5±34.5	1,2,3,4>6
RP	76.2±27.6	85.3±21.8	80.9±22.0	76.7±28.8	82.8±23.5	74.1±26.4	55.9±32.8	1>5; 1,2,3,4,5>6
BP	86.7±22.9	88.2±20.6	92.8±16.3	87.2±19.9	90.7±20.4	83.5±25.5	78.9±29.2	2,4>6
GH	52.3±25.8	55.4±24.6	49.9±22.6	53.6±27.1	57.9±24.6	52.0±26.0	43.0±27.7	1,4>6
VT	67.2±19.7	71.2±17.9	69.7±18.9	66.6±22.5	72.2±16.7	65.1±18.0	57.9±22.1	1,2,4>6
SF	81.5±28.6	92.1±18.2	84.8±26.3	81.3±27.4	88.6±24.0	78.7±29.4	61.5±34.7	1>5; 1,2,3,4,5>6
RE	89.3±19.4	91.9±16.2	91.7±14.8	91.6±17.8	91.4±18.7	88.4±19.4	80.3±25.7	1,2,3,4>6
MH	79.6±15.6	80.4±14.0	80.1±13.0	81.4±16.3	80.8±15.3	78.9±15.8	75.8±18.5	
PCS	46.9±10.6	49.7±9.0	49.0±8.7	46.5±10.7	49.9±8.4	45.8±10.9	40.4±12.5	1,2,3,4,5>6
MCS	57.2±8.0	58.3±6.7	57.4±7.7	58.1±7.4	58.0±7.3	56.9±7.9	54.3±10.2	1,3,4>6
**SF-6D**								
Health Preference	0.825±0.14	0.871±0.12	0.832±0.12	0.831±0.14	0.858±0.12	0.817±0.13	0.732±0.15	1,2,3,4,5>6

Note:

FACT-C subscales: PWB = physical well-being; SWB = social well-being; EWB = emotional well-being; FWB = functional well-being; CCS = colorectal cancer subscale; TOI = trial outcome index;

SF-12 subscales: PF = physical functioning; RP = role physical; BP = bodily pain; GH = general health; VT = vitality; SF = social functioning; RE = role emotional; MH = mental health; PCS = Physical Composite Summary; MCS = Mental Composite Summary

Higher score represents a higher level of functioning or better quality of life

* Significant difference between six colorectal neoplasm groups by Tukey's Post-hoc multiple comparisons. 1:Low Risk; 2:High Risk; 3:StageI; 4:StageII; 5:StageIII; 6:StageIV;

### Correlates of Generic and Health Preference Measures of HRQOL

Table 5 demonstrates the socio-demographic and clinical factors associated with generic and health preference measures of HRQOL by multiple linear regression models. Stage of disease at diagnosis was the clinical determinants for HRQOL outcomes with statistical significance in each indicator level of stage. Clinical and socio-demographic factors explained 11.9% (8.3%), 10.2% (6.6%) and 14.8% (11.3%) of the total variation, as determined by *R^2^* (adjusted *R^2^*), in PCS, MCS and health preference scores of patients with CRN, respectively. The multicollinearity did not occur in our regression models because the tolerance statistic and VIF were ≥0.1 and ≤10, respectively, for all significant regression coefficients. Health preference score was poorer in patients with rectal neoplasm (−0.0302, 95% CI: −0.0597 to −0.0008) but better in longer diagnosis duration (0.0002, 95% CI: 0.0000 to 0.0005). MCS and health preference scores were greater in patients who were male and older. Rectal neoplasm patients reported worse PCS scores (2.81, 95% CI: −5.14 to −0.47) compared to sigmoid neoplasm patients. HRQOL scores did not differ significantly by the presence of chronic co-morbidities and family history of colorectal cancer.

**Table 5:** Clinical and Socio-demographic Factors Associated with HRQOL Scores in CRN Patients (n = 515) by Multiple Linear Regression.

	SF-12 PCS			SF-12 MCS			SF-6D			Tolerance	VIF
Independent Variables	Coeff	S.E.	95% C.I.	Coeff	S.E.	95% C.I.	Coeff	S.E.	95% C.I.		
Constant	*39.81	3.78	(32.41,47.21)	*45.40	2.87	(39.78,51.01)	*0.6436	0.0478	(0.5500,0.7372)		
*Clinical Factors*											
Colorectal Neoplasm Staging (Stage IV)											
Low-risk Polyp	*8.44	1.66	(5.19,11.69)	*3.82	1.26	(1.35,6.28)	*0.1248	0.0210	(0.0837,0.1659)	0.506	1.975
High-risk Polyp	*8.02	1.73	(4.63,11.42)	2.14	1.31	(−0.44,4.71)	*0.0841	0.0219	(0.0411,0.1270)	0.572	1.748
Stage I	*5.49	1.70	(2.15,8.83)	*3.74	1.29	(1.21,6.28)	*0.0903	0.0215	(0.0481,0.1326)	0.504	1.986
Stage II	*8.91	1.59	(5.80,12.02)	*3.10	1.20	(0.74,5.46)	*0.1143	0.0201	(0.0749,0.1537)	0.490	2.040
Stage III	*4.89	1.54	(1.88,7.91)	*2.61	1.17	(0.33,4.90)	*0.0760	0.0195	(0.0379,0.1142)	0.486	2.058
Months Since Diagnosis†	0.02	0.01	(0.00,0.03)	0.00	0.01	(−0.01,0.01)	*0.0002	0.0001	(0.0000,0.0005)	0.868	1.152
Primary (Sigmoid)											
Colon	−1.74	1.20	(−4.10,0.62)	0.01	0.91	(−1.78,1.80)	−0.0073	0.0152	(−0.0372,0.0225)	0.564	1.773
Rectum	*−2.81	1.19	(−5.14, −0.47)	−1.15	0.90	(−2.91,0.62)	*−0.0302	0.0150	(−0.0597, −0.0008)	0.561	1.783
Family History of CRC	0.36	1.16	(−1.91,2.62)	−0.36	0.88	(−2.08,1.36)	0.0047	0.0146	(−0.0240,0.0333)	0.947	1.056
Chronic co-morbidities	−0.18	1.03	(−2.20,1.84)	−0.30	0.78	(−1.84,1.23)	−0.0090	0.0130	(−0.0346,0.0165)	0.797	1.255
*Socio-demographic Factors*											
Male	0.42	1.05	(−1.64,2.49)	*2.06	0.80	(0.49,3.62)	*0.0297	0.0133	(0.0036,0.0557)	0.714	1.401
Age†	0.04	0.05	(−0.06,0.14)	*0.16	0.04	(0.08,0.24)	*0.0014	0.0006	(0.0001,0.0027)	0.568	1.761
Education (Tertiary)											
No Formal Schooling	−0.33	2.01	(−4.28,3.61)	0.09	1.53	(−2.90,3.08)	0.0026	0.0254	(−0.0472,0.0525)	0.497	2.012
Primary	0.40	1.59	(−2.72,3.52)	−1.20	1.21	(−3.57,1.17)	0.0056	0.0201	(−0.0339,0.0450)	0.331	3.019
Secondary	−0.43	1.47	(−3.31,2.44)	−0.23	1.11	(−2.40,1.95)	0.0032	0.0185	(−0.0331,0.0395)	0.369	2.709
Married	0.04	1.07	(−2.06,2.15)	−0.09	0.82	(−1.68,1.51)	0.0046	0.0136	(−0.0221,0.0312)	0.904	1.106
Currently Working	1.55	1.29	(−0.97,4.08)	0.77	0.98	(−1.15,2.68)	0.0311	0.0163	(−0.0008,0.0630)	0.637	1.570
Household Monthly Income ≤HK$20000	−1.89	1.43	(−4.69,0.92)	−0.65	1.08	(−2.78,1.47)	−0.0213	0.0181	(−0.0568,0.0141)	0.688	1.453
Ever Smoking	0.75	1.19	(−1.59,3.09)	−1.11	0.91	(−2.89,0.66)	0.0209	0.0151	(−0.0087,0.0505)	0.686	1.458
Ever Drinking	0.26	1.18	(−2.05,2.57)	0.23	0.89	(−1.52,1.98)	−0.0095	0.0149	(−0.0386,0.0197)	0.706	1.417
R^2^	11.9%			10.2%			14.8%				
Adjusted R^2^	8.3%			6.6%			11.3%				

Note: CRN = Colorectal Neoplasms; CRC = Colorectal Cancer; VIF = Variance Inflation Factors; PCS = Physical Composite Summary; MCS = Mental Composite Summary

() Variable in brackets is the reference category for independent variables

* Significant independent variables (p<0.05) on HRQOL by multivariate linear regression;† HRQOL scores change in coefficient for each unit increase in independent variable.

## Discussion

This is the first study, to our knowledge, to compare the HRQOL in Chinese patients with CRN with the general population and to highlight the HRQOL differences among stage of disease at the time of diagnosis. CRN patients reported similar physical-related HRQOL and better mental-related HRQOL compared to matched controls from the Chinese general population. Among all socio-demographic and clinical factors, stage of disease at diagnosis was the only significant and influential factor correlated to generic and health preference scores of HRQOL outcomes. Compared to sigmoid neoplasms, rectal neoplasms were associated with poorer outcomes on generic and health preference although mental-related HRQOL showed a non-significant decline. The effect of time since diagnosis on HRQOL was positive but significant for health preference scores only. Socio-demographic factors such as educational level, marital status, working status, household income, smoking status and drinking status were not significant correlates of HRQOL outcomes.

Many studies collected the HRQOL of a sample of CRC patients and compared with normative values published in population-based data. Amongst comparative studies, only one UK study utilized the SF-12 scores comparisons of general population norms with colorectal cancer patients after surgery[24]. Those cancer survivors had lower PCS score and higher MCS score compared to the UK population with an age interval of 65–74 years. Our study found the signs of difference between groups were consistent with the UK study, regardless of physical-related and mental-related HRQOL. However, the present study did not detect significant differences in physical-related HRQOL between groups, indicating the CRN patients were not associated with significant impairment of the physical-related HRQOL in Chinese population.

HRQOL decreased linearly with more severe stages of colorectal cancer, providing consistent evidence as reported in European and Australian studies[25]–[27]. On the other hand, based on the data presented in the current study, patients with stage II colorectal cancer reported better HRQOL than those with stage I colorectal cancer but worse HRQOL than those with advanced stage of cancer. These particular findings were in line with two previous US studies which assessed the health preference scores using the Health Utility Index Mark III (HUI3) among colorectal cancer patients with 13–24 months from diagnosis[3]; and assessed the vitality subscale using the SF-36 Health Survey among long term colorectal cancer survivors who were at least five years from initial diagnosis[4]. However, no evidence from the US studies [3], [4], [28]–[30] had a tendency of relationship between stage at diagnosis and HRQOL. One of the possible reasons was, in turn, the change in the perception (or re-conceptualization) of HRQOL so called response shift [31], as a result of either clinical interventions or self-coping with colorectal cancer[32].

The present study suggested that factors associated with HRQOL scores were different from those related to health preference scores. Significant improvements in patients' HRQOL scores, but surprising not in health preference scores, were found with longer time since CRN diagnosis. Ramsey et al. [3] addressed that strong and negative impact on health preference measure was experienced within the first two years of colorectal cancer diagnosis but the uniform improvement over two years was found. There was no significant change detected among different time periods in patients who were at least 13 months [3] and at least 60 months (or 5 years) [4] from initial diagnosis of colorectal cancer. However, longer time since diagnosis was associated with better health preference measure. It is postulated that the inconsistent findings between previous and current studies were in part due to the differences in the measurement type of ‘Time from Diagnosis’. Those studies conducted data analysis by partitioning ‘Time from Diagnosis’ into several time periods as an ordinal factor.

Physical aspect of HRQOL and health preference scores reported by rectal neoplasm patients were significantly worse than those scores reported by patients with other primary tumour locations. It is possible that the impact of tumour site on HRQOL was primarily due to the effects of treatment modalities. Rectal neoplasm may be undertaken by the radiotherapy before or after surgery but colon neoplasm does not. Most of the patients with rectal cancer underwent resection with the installation of stoma [10], leading to the burden of daily activities and living. Having stoma was significantly associated with lower HRQOL scores in Western populations (e.g. France [33], US [34], [35] and Denmark[36]). A Japanese study [37] showed that patients with stoma had worse health preference scores derived using direct valuation techniques. No significant HRQOL difference among patients with stoma was found in our Chinese population, which paralleled with the most recent study on colorectal cancer survivors[38]. One interpretation of our findings was that HRQOL of patients with stoma was improved by the growing medical advances in surgical resection and follow-up stoma care services, and further reduced the risk of stoma complications and identified better adaptation and coping strategy to live with stoma [39].

### Limitations

Several shortcomings were noted. First, sampling bias regarding the use of convenience sampling from a specialist outpatient clinic of one regional hospital in Hong Kong may limit the generalizability of results to CRN patients in Chinese and other populations. A high proportion of unemployed, low household income, or low educated patients in this sample has raised caution with interpretation and extrapolation the findings to other populations with different patient characteristics. Moreover, possible sampling issue related to non-response bias was addressed in a previous study because subjects who did not respond in follow-up assessment had a significant inferior HRQOL at the initial survey than those who did[40]; healthier patients seemed less likely to be dropped from the follow-up assessments. In such circumstance, non-response bias led by the dropout of consented subjects with inferior HRQOL may potentially underestimate the HRQOL in this study. Second, *R^2^* (or adjusted *R^2^*) did not exceed 20% in linear regression analyses, suggesting that only a small proportion of the variance in HRQOL outcomes could be explained by the independent variables. The inclusion of variables related to adiposity and healthy lifestyle behaviours such as consumption of fruit and vegetable, and regular exercise, was an attempt to increase the understanding of underlying factors associated with HRQOL. Finally, as a cross-sectional study, our results can only give clues on casual and effect relationships but not the definitive relationships between independent variables and HRQOL.

## Conclusion

Stage of disease at initial diagnosis was the most significant clinical correlate for all HRQOL outcomes in patients with CRN. Specificity, those CRN patients who simultaneously had rectum as primary tumor site and severe stage of disease at diagnosis, indicated suboptimal HRQOL in relation to physical aspect of HRQOL and health preference scores. Based on available Chinese data on health preference scores, the QALYs is estimated for the cost-effectiveness analysis of CRN-related interventions in economics evaluation.

## References

[1] Cella DF, Tulsky DS (1993) Quality of Life in Cancer: Definition, Purpose, and Method of Measurement. Cancer Investigation 11: 327–336. 8485655 10.3109/07357909309024860

[2] Byrne C, Griffin A, Blazeby J, Conroy T, Efficace F (2007) Health-related quality of life as a valid outcome in the treatment of advanced colorectal cancer. European Journal of Surgical Oncology 33: S95–S104. 18039559 10.1016/j.ejso.2007.10.003

[3] Ramsey SD, Andersen MR, Etzioni R, Moinpour C, Peacock S, et al (2000) Quality of life in survivors of colorectal carcinoma. Cancer 88: 1294–1303. 10717609

[4] Ramsey SD, Berry K, Moinpour C, Giedzinska A, Andersen MR (2002) Quality of life in long term survivors of colorectal cancer. The American Journal of Gastroenterology 97: 1228–1234. 12017152 10.1111/j.1572-0241.2002.05694.x

[5] Simon AE, Thompson MR, Flashman K, Wardle J (2009) Disease stage and psychosocial outcomes in colorectal cancer. Colorectal Disease 11: 19–25. 18355377 10.1111/j.1463-1318.2008.01501.x

[6] National Institute for Clinical Excellence (2008) Guide to the Methods of Technology Appraisal (reference N1618). London: NICE.

[7] Atkin WS, Saunders BP (2002) Surveillance guidelines after removal of colorectal adenomatous polyps. Gut 51: v6–9. 12221031 10.1136/gut.51.suppl_5.v6 PMC1867736

[8] Greene FL, Page DL, Fleming ID, Balch CM, Haller DG, et al.. (2002) AJCC Cancer Staging Manual. New York: Springer Publishers.

[9] Wong CKH, Lam CLK, Rowen D, McGhee SM, Ma KP, et al (2012) Mapping the Functional Assessment of Cancer Therapy-General or -Colorectal to SF-6D in Chinese Patients with Colorectal Neoplasm. Value in Health 15: 495–503. 22583460 10.1016/j.jval.2011.12.009

[10] Wong CKH, Lam CLK, Poon JTC, McGhee SM, Law WL, et al (2012) Direct Medical Costs of Care for Chinese Patients with Colorectal Neoplasia: a Health Care Service Provider Perspective. Journal of Evaluation in Clinical Practice 18: 1203–1210. 22111837 10.1111/j.1365-2753.2011.01776.x

[11] Wong CKH, Lam CLK, Mulhern B, Law WL, Poon JTC, et al (2012) Measurement Invariance of the Functional Assessment of Cancer Therapy-Colorectal Quality-of-life Instrument among Modes of Administration. Quality of Life Research In Press DOI:10.1007/s11136-012-0272-x. 10.1007/s11136-012-0272-x PMC3731518 23054490

[12] Wong CKH, Lam CLK, Law WL, Poon JTC, Kwong DLW, et al (2013) Condition-specific Measure was more responsive than Generic Measure in Colorectal Cancer: All but Social Domains. Journal of Clinical Epidemiology In Press DOI:10.1016/j.jclinepi.2012.11.010. 10.1016/j.jclinepi.2012.11.010 23548135

[13] Wong CKH, Lam CLK, Law WL, Poon JTC, Chan P, et al (2012) Validity and Reliability Study on Traditional Chinese FACT-C in Chinese Patients with Colorectal Neoplasm. Journal of Evaluation in Clinical Practice 18: 1186–1195. 21851512 10.1111/j.1365-2753.2011.01753.x

[14] Wong CKH, Lam CLK, Wan YF, Rowen D (2013) Predicting SF-6D from the European Organization for Treatment and Research of Cancer Quality of Life Questionnaire Scores in Patients with Colorectal Cancer. Value in Health 16: 353–364. 10.1016/j.jval.2012.12.004 23538190

[15] Lam CLK, Wong CKH, Lam ETP, Lo YYC, Huang WW (2010) Population Norm of Chinese (HK) SF-12 Health Survey_Version 2 of Chinese Adults in Hong Kong. Hong Kong Practitioner 32: 77–86.

[16] Lam CLK, Tse EYY, Gandek B (2005) Is the standard SF-12 Health Survey valid and equivalent for a Chinese population? Quality of Life Research 14: 539–547. 15892443 10.1007/s11136-004-0704-3

[17] Fong DYT, Lam CLK, Mak KK, Lo WS, Lai YK, et al (2010) The Short Form-12 Health Survey was a valid instrument in Chinese adolescents. Journal of Clinical Epidemiology 63: 1020–1029. 20189764 10.1016/j.jclinepi.2009.11.011

[18] Lam CLK, Brazier J, McGhee SM (2008) Valuation of the SF-6D Health States Is Feasible, Acceptable, Reliable, and Valid in a Chinese Population. Value in Health 11: 295–303. 18380642 10.1111/j.1524-4733.2007.00233.x

[19] Lam ETP, Lam CLK, Fong DYT, Huang WW (2013) Is the SF-12 version 2 Health Survey a valid and equivalent substitute for the SF-36 version 2 Health Survey for the Chinese? Journal of Evaluation in Clinical Practice 19: 200–208. 22128754 10.1111/j.1365-2753.2011.01800.x

[20] Brazier JE, Roberts J (2004) The Estimation of a Preference-Based Measure of Health From the SF-12. Medical Care 42: 851–859. 15319610 10.1097/01.mlr.0000135827.18610.0d

[21] Brazier J, Roberts J, Deverill M (2002) The estimation of a preference-based measure of health from the SF-36. Journal of Health Economics 21: 271–292. 11939242 10.1016/s0167-6296(01)00130-8

[22] McGhee SM, Brazier J, Lam CLK, Wong LC, Chau J, et al (2011) Quality-adjusted life years: population-specific measurement of the quality component. Hong Kong Medical Journal 17 suppl 617–21. 22147354

[23] Hays RD, Morales LS (2001) The RAND-36 measure of health-related quality of life. Annals of Medicine 33: 350–357. 11491194 10.3109/07853890109002089

[24] Wilson T, Alexander D, Kind P (2006) Measurement of Health-Related Quality of Life in the Early Follow-Up of Colon and Rectal Cancer. Diseases of the Colon & Rectum 49: 1692–1702. 17041750 10.1007/s10350-006-0709-9

[25] Sharma A, Sharp DM, Walker LG, Monson JRT (2007) Predictors of early postoperative quality of life after elective resection for colorectal cancer. Annals of Surgical Oncology 14: 3435–3442. 17896155 10.1245/s10434-007-9554-x

[26] Paika V, Almyroudi A, Tomenson B, Creed F, Kampletsas EO, et al (2010) Personality variables are associated with colorectal cancer patients' quality of life independent of psychological distress and disease severity. Psycho-Oncology 19: 273–282. 19353527 10.1002/pon.1563

[27] Rinaldis M, Pakenham KI, Lynch BM (2010) Relationships between quality of life and finding benefits in a diagnosis of colorectal cancer. British Journal of Psychology 101: 259–275. 19519986 10.1348/000712609X448676

[28] Trentham-Dietz A, Remington PL, Moinpour CM, Hampton JM, Sapp AL, et al (2003) Health-Related Quality of Life in Female Long-Term Colorectal Cancer Survivors. Oncologist 8: 342–349. 12897331 10.1634/theoncologist.8-4-342

[29] Sultan S, Fisher DA, Voils CI, Kinney AY, Sandler RS, et al (2004) Impact of functional support on health-related quality of life in patients with colorectal cancer. Cancer 101: 2737–2743. 15536617 10.1002/cncr.20699

[30] Ristvedt SL, Trinkaus KM (2009) Trait anxiety as an independent predictor of poor health-related quality of life and post-traumatic stress symptoms in rectal cancer. British Journal of Health Psychology 14: 701–715. 19171084 10.1348/135910708X400462 PMC2756319

[31] Sprangers MAG, Schwartz CE (1999) Integrating response shift into health-related quality of life research: a theoretical model. Social Science & Medicine 48: 1507–1515. 10400253 10.1016/s0277-9536(99)00045-3

[32] Bernhard J, Hürny C, Maibach R, Herrmann R, Laffer U, et al (1999) Quality of life as subjective experience: Reframing of perception in patients with colon cancer undergoing radical resection with or without adjuvant chemotherapy. Annals of Oncology 10: 775–782. 10470423 10.1023/a:1008311918967

[33] Rauch P, Miny J, Conroy T, Neyton L, Guillemin F (2004) Quality of Life Among Disease-Free Survivors of Rectal Cancer. Journal of Clinical Oncology 22: 354–360. 14722043 10.1200/JCO.2004.03.137

[34] Liu L, Herrinton LJ, Hornbrook MC, Wendel CS, Grant M, et al (2010) Early and Late Complications Among Long-Term Colorectal Cancer Survivors With Ostomy or Anastomosis. Diseases of the Colon & Rectum 53: 200–212. 20087096 10.1007/DCR.0b013e3181bdc408 PMC3320086

[35] Krouse RS, Herrinton LJ, Grant M, Wendel CS, Green SB, et al (2009) Health-Related Quality of Life Among Long-Term Rectal Cancer Survivors With an Ostomy: Manifestations by Sex. Journal of Clinical Oncology 27: 4664–4670. 19720920 10.1200/JCO.2008.20.9502 PMC2754912

[36] Ross L, Abild-Nielsen A, Thomsen B, Karlsen R, Boesen E, et al (2007) Quality of life of Danish colorectal cancer patients with and without a stoma. Supportive Care in Cancer 15: 505–513. 17103196 10.1007/s00520-006-0177-8

[37] Shiroiwa T, Fukuda T, Tsutani K (2009) Health utility scores of colorectal cancer based on societal preference in Japan. Quality of Life Research 18: 1095–1103. 19626462 10.1007/s11136-009-9513-z

[38] Hornbrook MC, Wendel CS, Coons SJ, Grant M, Herrinton LJ, et al (2011) Complications Among Colorectal Cancer Survivors: SF-6D Preference-Weighted Quality of Life Scores. Medical Care 49: 321–326. 21224741 10.1097/MLR.0b013e31820194c8 PMC3503529

[39] de Gouveia Santos VLC, Chaves EC, Kimura M (2006) Quality of Life and Coping of Persons With Temporary and Permanent Stomas. Journal of Wound Ostomy & Continence Nursing 33: 503–509. 10.1097/00152192-200609000-00008 17133138

[40] Yost K, Hahn E, Zaslavsky A, Ayanian J, West D (2008) Predictors of health-related quality of life in patients with colorectal cancer. Health and Quality of Life Outcomes 6: 66. 18724874 10.1186/1477-7525-6-66 PMC2538505

